# Image quality improvement with deep learning‐based reconstruction on abdominal ultrahigh‐resolution CT: A phantom study

**DOI:** 10.1002/acm2.13318

**Published:** 2021-06-23

**Authors:** Takashi Shirasaka, Tsukasa Kojima, Yoshinori Funama, Yuki Sakai, Masatoshi Kondo, Ryoji Mikayama, Hiroshi Hamasaki, Toyoyuki Kato, Yasuhiro Ushijima, Yoshiki Asayama, Akihiro Nishie

**Affiliations:** ^1^ Division of Radiology Department of Medical Technology Kyushu University Hospital Fukuoka Japan; ^2^ Department of Medical Physics Faculty of Life Sciences Kumamoto University Kumamoto Japan; ^3^ Department of Clinical Radiology Graduate School of Medical Sciences Kyushu University Fukuoka Japan; ^4^ Department of Advanced Imaging and Interventional Radiology Graduate School of Medical Sciences Kyushu University Fukuoka Japan

**Keywords:** deep learning‐based reconstruction, ultrahigh‐resolution CT

## Abstract

**Purpose:**

In an ultrahigh‐resolution CT (U‐HRCT), deep learning‐based reconstruction (DLR) is expected to drastically reduce image noise without degrading spatial resolution. We assessed a new algorithm's effect on image quality at different radiation doses assuming an abdominal CT protocol.

**Methods:**

For the normal‐sized abdominal models, a Catphan 600 was scanned by U‐HRCT with 100%, 50%, and 25% radiation doses. In all acquisitions, DLR was compared to model‐based iterative reconstruction (MBIR), filtered back projection (FBP), and hybrid iterative reconstruction (HIR). For the quantitative assessment, we compared image noise, which was defined as the standard deviation of the CT number, and spatial resolution among all reconstruction algorithms.

**Results:**

Deep learning‐based reconstruction yielded lower image noise than FBP and HIR at each radiation dose. DLR yielded higher image noise than MBIR at the 100% and 50% radiation doses (100%, 50%, DLR: 15.4, 16.9 vs MBIR: 10.2, 15.6 Hounsfield units: HU). However, at the 25% radiation dose, the image noise in DLR was lower than that in MBIR (16.7 vs. 26.6 HU). The spatial frequency at 10% of the modulation transfer function (MTF) in DLR was 1.0 cycles/mm, slightly lower than that in MBIR (1.05 cycles/mm) at the 100% radiation dose. Even when the radiation dose decreased, the spatial frequency at 10% of the MTF of DLR did not change significantly (50% and 25% doses, 0.98 and 0.99 cycles/mm, respectively).

**Conclusion:**

Deep learning‐based reconstruction performs more consistently at decreasing dose in abdominal ultrahigh‐resolution CT compared to all other commercially available reconstruction algorithms evaluated.

## 1 INTRODUCTION

An ultrahigh‐resolution CT (U‐HRCT) scanner became available for clinical practice in 2017, and several studies have reported its advantages.^1^, ^2^, ^3^, ^4^, ^5^, ^6^ However, the increased image noise that occurs when U‐HRCT is applied (along with the improved spatial resolution) is a common concern,^1^, ^3^, ^4^, ^5^, ^6^, ^7^, ^8^ because the amount of image noise is affected by the slice thickness and matrix size. Abdominal dynamic CT is an essential imaging modality for malignant liver tumors such as hepatocellular carcinoma, cholangiocellular carcinoma, and metastatic tumors.^9^, ^10^, ^11^ U‐HRCT is expected to demonstrate tiny vessels and pathological conditions in greater detail. With U‐HRCT, it is difficult to successfully control the appropriate radiation dose because the tube current is limited by the combination of focal spot size and exposure time. In addition, for multiphase scanning, it is necessary to minimize the radiation dose in each phase while maintaining a diagnostically adequate image quality.^12^, ^13^, ^14^, ^15^ Although model‐based iterative reconstruction (MBIR) is used to reduce the image noise that accompanies an insufficient radiation dose, the use of MBIR requires considerable computational time for image reconstruction,^16^ which can affect clinical practice.

A deep learning‐based reconstruction (DLR) algorithm was recently released in U‐HRCT and is expected to reduce image noise dramatically without degrading spatial resolution.^16^, ^17^ Compared to MBIR, DLR can reconstruct images more quickly and is expected to minimize a change in noise texture that is specific to the iterative reconstruction derived from a low radiation dose or the level of iterative reconstruction.^18^, ^19^, ^20^ Therefore, we evaluated DLR in abdominal dynamic CT using U‐HRCT. In the present study, we assessed the image noise and spatial resolution characteristics of the DLR algorithm at different radiation doses on abdominal U‐HRCT compared with filtered back projection (FBP), hybrid iterative reconstruction (HIR), and MBIR.

## 2 METHODS

Our present study was performed with a phantom imaging experiment. Therefore, there was no need for institutional review board approval.

### 2.1 DLR algorithm

DLR incorporates a deep convolutional neural networks (DCNN) restoration process into the reconstruction flow. For the deep learning‐based approach, given HIR images and high‐dose MBIR images as training pairs, statistical features that differentiate signal from the noise and artifacts could be “learned” in the training process and then be “updated” in the DCNN kernel for future inference use. Millions of image pairs were used in the training of DLR. The gold standard clinical reference images were acquired with high tube current and reconstructed with true MBIR, and the true MBIR used a greater number of iterations than could be otherwise used in a clinical setting due to time constraints. No phantom data were included in the gold standard reference images. This training process was previously completed during the development phase with no off‐site unsupervised training—which could alter the algorithm performance—taking place.

### 2.2 Body phantom

**Fig. 1:**
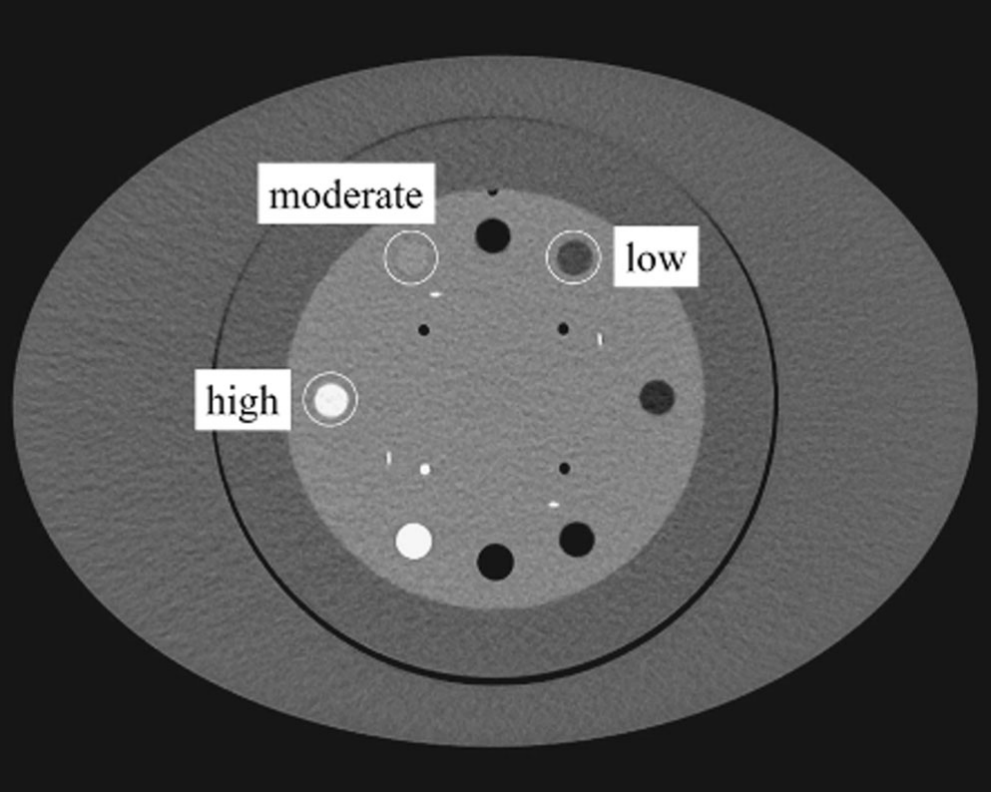
Axial image of the body phantom. A Catphan phantom attached to an oval annulus (25 × 35 cm; 95‐cm circumference). The CTP 404, 486, and 515 modules were used to assess the in‐plane spatial resolution, the image noise, and the low contrast detectability, respectively. The high‐ (estimated value of 340 HU), moderate‐ (estimated value of 120 HU), and low‐ (estimated value of −35 HU) signal objects were located at 9, 11, and 1 o'clock, respectively.

For normal‐sized abdominal models, we used the Catphan 600 (The Phantom Laboratory, Salem, NY, USA) attached with an oval annulus (25 × 35 cm; 95‐cm circumference, Fig. 1). We used three different modules, CTP 404, CTP 486, and CTP 515, for the image assessment.

### 2.3 CT scanning

The phantom was scanned using the U‐HRCT scanner (Aquilion Precision; Canon Medical Systems, Otawara, Japan). This scanner has three scan modes: normal‐, high‐, and superhigh‐resolution modes.^5^ In the present study, we used the high‐resolution mode with an 80‐row detector configuration of 0.5 mm detectors (1792 channels). Image reconstruction was performed with a 1024 × 1024 matrix size. The nominal focal spot size of the X‐ray tube was 0.9 × 1.2 mm, which was equivalent to the small focus of the conventional area detector CT (Aquilion ONE ViSION edition; Canon Medical Systems, Otawara, Japan). The combination of scan mode and focal spot size was selected assuming abdominal dynamic CT because the selected focal spot size limits the maximum tube current and exposure time. The other imaging parameters were as follows:

Tube voltage was set to 120 kVp and the rotation time was set to 0.5 s. Tube current was varied from 590 to 300 to 150 mA as 100%, 50%, and 25% radiation doses, respectively. Images were reconstructed at a 0.5‐mm thickness with a 400 mm of field of view. For evaluation of low contrast detectability, images were reconstructed at a 5‐mm thickness. In all acquisitions, DLR (Advanced Intelligent Clear‐IQ Engine [AiCE], Canon Medical Systems) with a clinically optimized body parameter, “body standard” and FBP reconstruction with the FC13 kernel were performed. In addition, two types of iterative reconstruction were conducted: HIR with the FC13 kernel, and MBIR reconstruction (AIDR 3D standard and FIRST body standard, respectively; Canon Medical Systems) (Table 1).

**Table 1:** CT scanning and reconstruction settings for the four algorithms.

	FBP	HIR	MBIR		DLR
Acquisition mode	High‐resolution mode (1,792 channels)				
Focal spot size	0.9 × 1.2 mm				
Tube voltage	120 kVp				
Tube current (CTDIvol)	590, 300, 150‐mA (26.3, 13.4, 6.7‐mGy)				
Rotation time	0.5 s				
Collimation (configuration)	40 mm (80 × 0.5 mm)				
Pitch	0.8				
Field of view	400 mm				
Slice thickness	0.5 mm, 5 mm				
Image matrix	1024 × 1024				
Kernel/parameter	FC13	FC13/standard	Body standard	Body standard	

Abbreviations: CTDIvol, volume computed tomography dose index; DLR, deep learning‐based reconstruction; FBP, filtered back projection; HIR, hybrid iterative reconstruction; MBIR, model‐based iterative reconstruction.

### 2.4 Image assessment

For the quantitative assessment, the CT number, image noise, frequency characteristics of the image noise, signal visibility, and spatial resolution in all of the reconstruction algorithms were compared using Image J 1.52a (National Institutes of Health, Bethesda, MD, USA) and Excel 2016 (Microsoft, Redmond, WA). These quantitative analyses were obtained from one scan series. For the assessment of the low contrast detectability, a visual evaluation was performed by two radiologists.

#### 2.4.1 CT number

**Fig. 2:**
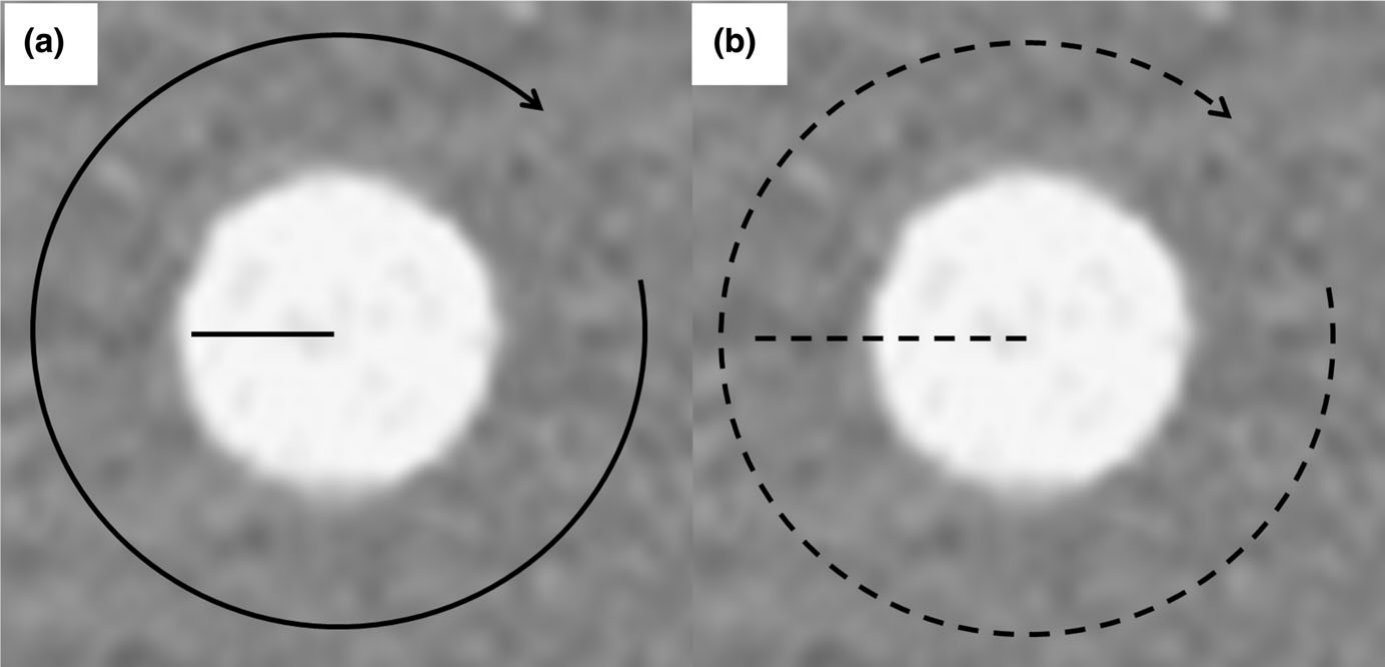
Alignment of the line ROI to record the CT number and attenuation profile curves. The CTP 404 module was used to record the CT number and attenuation profile curves of the high‐, moderate‐, and low‐signal objects. (a) The CT numbers of these objects for each reconstruction algorithm were recorded radially around the object centers at 1° intervals using a line region of interest (1 × 27 pixels, 5‐mm circle‐radius). (b) The attenuation profile curves of these objects for each reconstruction algorithm were recorded radially around the object centers at 1° intervals using a line region of interest (1 × 51 pixels, radius of the circle of 10 mm).

The CT numbers obtained with each reconstruction algorithm in the phantom experiments were compared using the CTP 404 module.^21^ Three different disk‐shaped objects with a diameter of 12 mm were used as the assumed abdominal structures (lumen of a contrast‐enhanced aorta, contrast‐enhanced tumor, and adipose tissue^22^). We used a delrin rod (estimated value of 340 Hounsfield units: HU), an acrylic rod (estimated value of 120 HU), and a polystyrene rod (estimated value of −35 HU) as the high‐, moderate‐, and low‐signal objects, respectively (Fig. 1). The CT numbers of these objects for each reconstruction algorithm were recorded radially around the object centers at 1° intervals using a line region of interest (ROI) (1 × 27 pixels, 5 mm circle‐radius). Mean CT numbers were obtained using the individual line ROI measurements [Fig. 2(a)].

#### 2.4.2 Image noise magnitude

The standard deviation (SD) of the CT number was defined as the image noise in the axial image. A square ROI (256 × 256 pixels) was placed at the center of each axial image of the CTP 486 module. We calculated the mean image noise magnitude using 50 sequential images.

#### 2.4.3 Noise power spectrum

To evaluate the frequency characteristics of the image noise, we calculated the noise power spectrum (NPS) by the radial frequency method^23^ using the CTP 486 module. The NPS curve was obtained from the center (256 × 256 pixels) ROIs used to analyze image noise. We also normalized the NPS by dividing the NPS value by the area‐under‐the‐curve of the NPS.

#### 2.4.4 Low contrast detectability

**Fig. 3:**
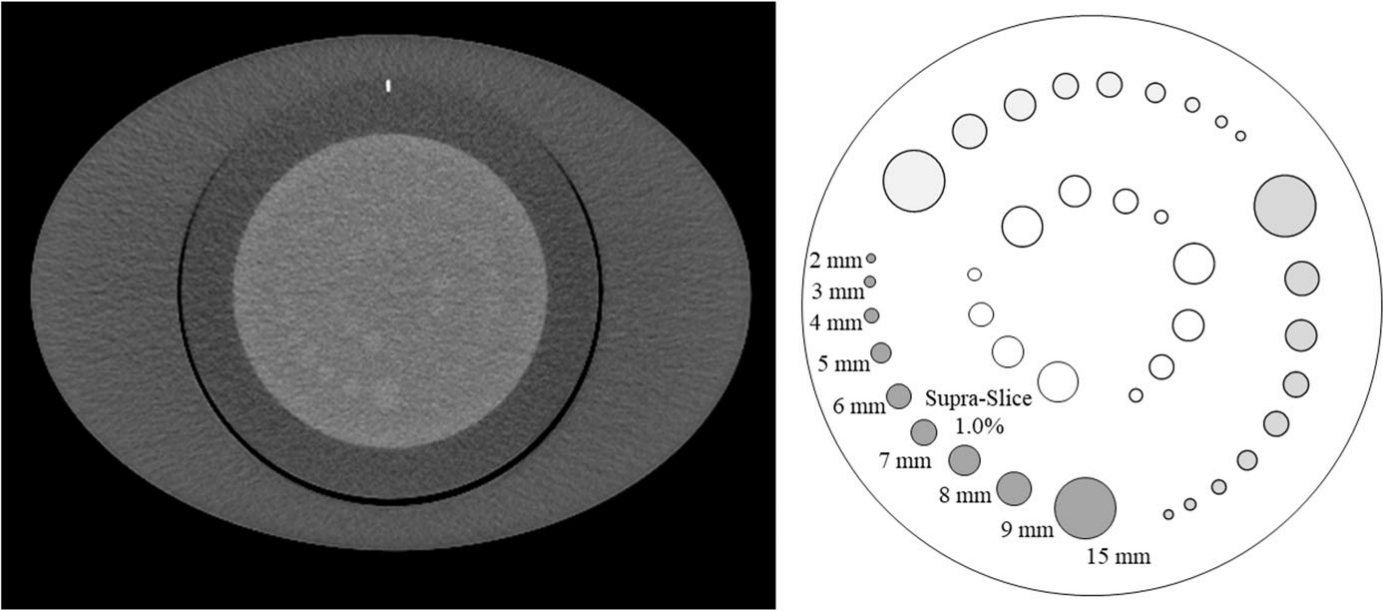
Axial image and schema of the low‐contrast objects (CTP515 module). Two observers recorded the detectable minimum diameter of the low‐contrast object. The diameters of the low‐contrast object were 15, 9, 8, 7, 6, 5, 4, 3, and 2 mm at the 1.0% contrast level.

Two board‐certified radiologists (Y.U. and A.N.) with 22 and 27 years of experience in abdominal radiology, respectively, and blinded to the radiation dose and reconstruction method independently evaluated the low contrast detectability using axial images of the CTP 515 module (Fig. 3). The diameters of the low contrast object at the 1.0% contrast level were 15, 9, 8, 7, 6, 5, 4, 3, and 2 mm. Each observer recorded the detectable minimum diameter of the low contrast object. Sixty images (three radiation doses × five scans × four types of reconstruction algorithms) were presented in random order to the two observers. Images were displayed with a window level and width of 60 and 250 HU, respectively. The low contrast detectability for each reconstruction algorithm was the median of the diameters reported by the two observers.

#### 2.4.5 Signal visibility

For the evaluation of the visibility of signals on axial images, attenuation profile curves (APCs) for all reconstruction algorithms were compared using the CTP 404 module. To obtain the mean APCs of the high‐, moderate‐, and low‐signal objects for each reconstruction algorithm, we recorded 360 APCs radially around the object's center at 1° intervals using a line ROI [1 × 51 pixels, 10 mm circle‐radius; Fig. 2(b)].

#### 2.4.6 Modulation transfer function

For in‐plane spatial resolution, modulation transfer function (MTF) curves for all reconstruction algorithms were calculated from the phantom experiments with the CTP 404 module. The MTF curves were calculated using an inserted disk‐shaped object (Teflon, estimated value of 990 HU) surrounded by a square ROI according to the disk methodology.^24^ First, a signal‐averaging image was generated from the 50 sequential images to reduce image noise. Ten consecutive signal‐averaging images reconstructed at 0.1‐mm intervals were used to obtain the mean MTF value. Then, the edge of the object was analyzed to determine the edge‐spread function, which was differentiated to obtain the line‐spread function. Finally, an object‐specific MTF was generated by Fourier transformation of the line‐spread function.

### 2.5 Statistical analysis

Data of the CT number are expressed as the mean ± standard deviation (SD). Data of the image noise magnitude and spatial resolution are expressed as the mean ± standard error (SE). The Cohen’s kappa test was used to assess the degree of agreement between the observers, with a kappa value of 0.01–0.20 for slight agreement, 0.21–0.40 for fair, 0.41–0.60 for moderate, 0.61–0.80 for substantial and 0.81–1.00 for almost perfect agreement. These analyses were performed using GraphPad Prism, version 7.01 (GraphPad Software, La Jolla, CA, USA).

## 3 RESULTS

### 3.1 CT number and image noise magnitude

**Fig. 4:**
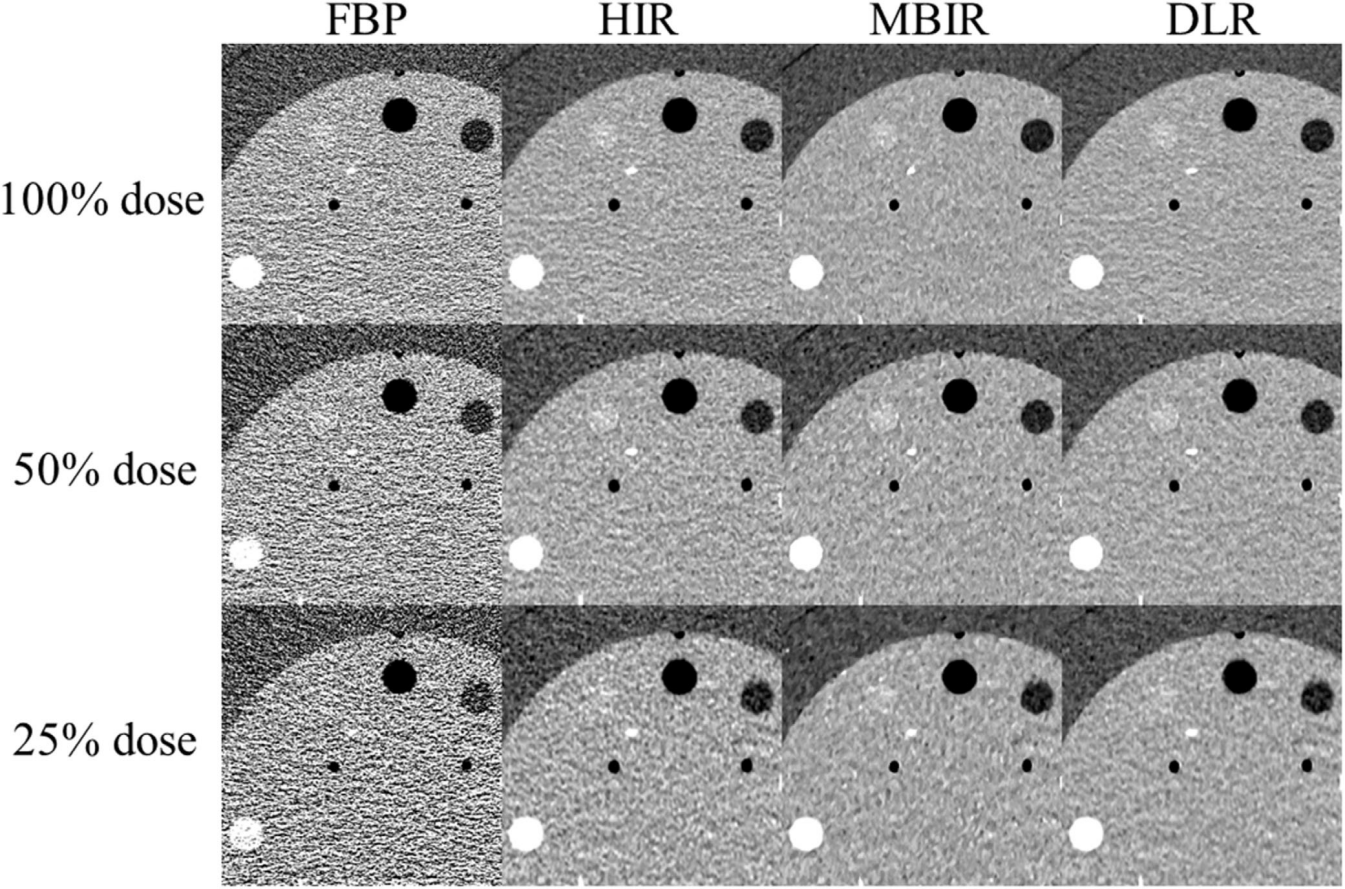
The clipped axial images (CTP404 module) of each reconstruction algorithm obtained at the 100%, 50%, and 25% radiation doses. As assumed abdominal structures (lumen of a contrast‐enhanced aorta, contrast‐enhanced tumor, and adipose tissue), three different disk‐shaped objects (high, moderate, and low) with a 12‐mm dia. were used to evaluate the signal visibility.

Figure 4 shows the clipped axial images of each reconstruction algorithm obtained at the different radiation doses. Although the CT number of the signal object part was slightly affected by the reconstruction algorithm that was used, all of the CT numbers obtained with all of reconstruction algorithms were similar (Table 2). With the MBIR, the CT number of the high‐signal object was slightly lower than the estimated value of 340 HU (100%, 50%, and 25% radiation doses: 318.5, 320.3, and 319 HU, respectively).

**Table 2:** CT number in each reconstruction algorithm at different radiation doses.

Radiation dose		Contrast	Reconstruction algorithm			
			FBP	HIR	MBIR	DLR
CT number	100%	High (340 HU)	333.4 ± 16.3	330.4 ± 8.7	318.5 ± 16.4	337.2 ± 7.7
		Moderate (120 HU)	124.1 ± 16.1	121.7 ± 8.1	121.0 ± 6.4	120.1 ± 6.5
		Low (−35 HU)	−35.4 ± 14.8	−31.6 ± 11	−32.8 ± 8.8	−34.8 ± 10.4
	50%	High (340 HU)	335.9 ± 33	334.6 ± 14.3	320.3 ± 16.9	342.7 ± 12.4
		Moderate (120 HU)	112.8 ± 32.6	118.3 ± 10.7	119.2 ± 7.5	116.6 ± 7.2
		Low (−35 HU)	−33 ± 17.3	−32.6 ± 7.7	−33.8 ± 6.5	−34.6 ± 6.5
	25%	High (340 HU)	335.9 ± 45.8	327.7 ± 9.7	319 ± 10.5	334.5 ± 11
		Moderate (120 HU)	125.3 ± 36.7	124.2 ± 11.5	126.2 ± 10.7	120.9 ± 8.1
		Low (−35 HU)	−39.3 ± 34.8	−28.9 ± 8.2	−31.8 ± 6.7	−30.6 ± 7.5

Abbreviations: DLR, deep learning‐based reconstruction; FBP, filtered back projection; HIR, hybrid iterative reconstruction; HU, Hounsfield units; MBIR, model‐based iterative reconstruction.

Table 3 shows the image noise magnitude of each reconstruction algorithm at each relative radiation dose. As the radiation dose decreased, the image noise magnitude of MBIR increased. The image noise magnitude was 153% (15.6 HU) for the 50% dose and 261% (26.6 HU) for the 25% dose compared to that of the 100% radiation dose (10.2 HU), respectively. In contrast, the image noise magnitude of DLR did not increase substantially with the decrease in the radiation dose. The image noise magnitude was 110% (16.9 HU) for the 50% dose and 108% (16.7 HU) for the 25% dose compared to that of the 100% radiation dose (15.4 HU), respectively.

**Table 3:** Image noise in each reconstruction algorithm at different radiation doses.

	Radiation dose	Reconstruction algorithm			
		FBP	HIR	MBIR	DLR
Image noise	100%	54.0 (0.09)	19.3 (0.05)	10.2 (0.12)	15.4 (0.06)
	50%	76.8 (0.17)	22.7 (0.05)	15.6 (0.09)	16.9 (0.09)
	25%	115.7 (0.23)	24.2 (0.03)	26.6 (0.05)	16.7 (0.04)
Hounsfield units (SE)					

Abbreviations: DLR, deep learning‐based reconstruction; FBP, filtered back projection; HIR, hybrid iterative reconstruction; MBIR, model‐based iterative reconstruction; SE, standard error.

### 3.2 Noise power spectrum

**Fig. 5:**
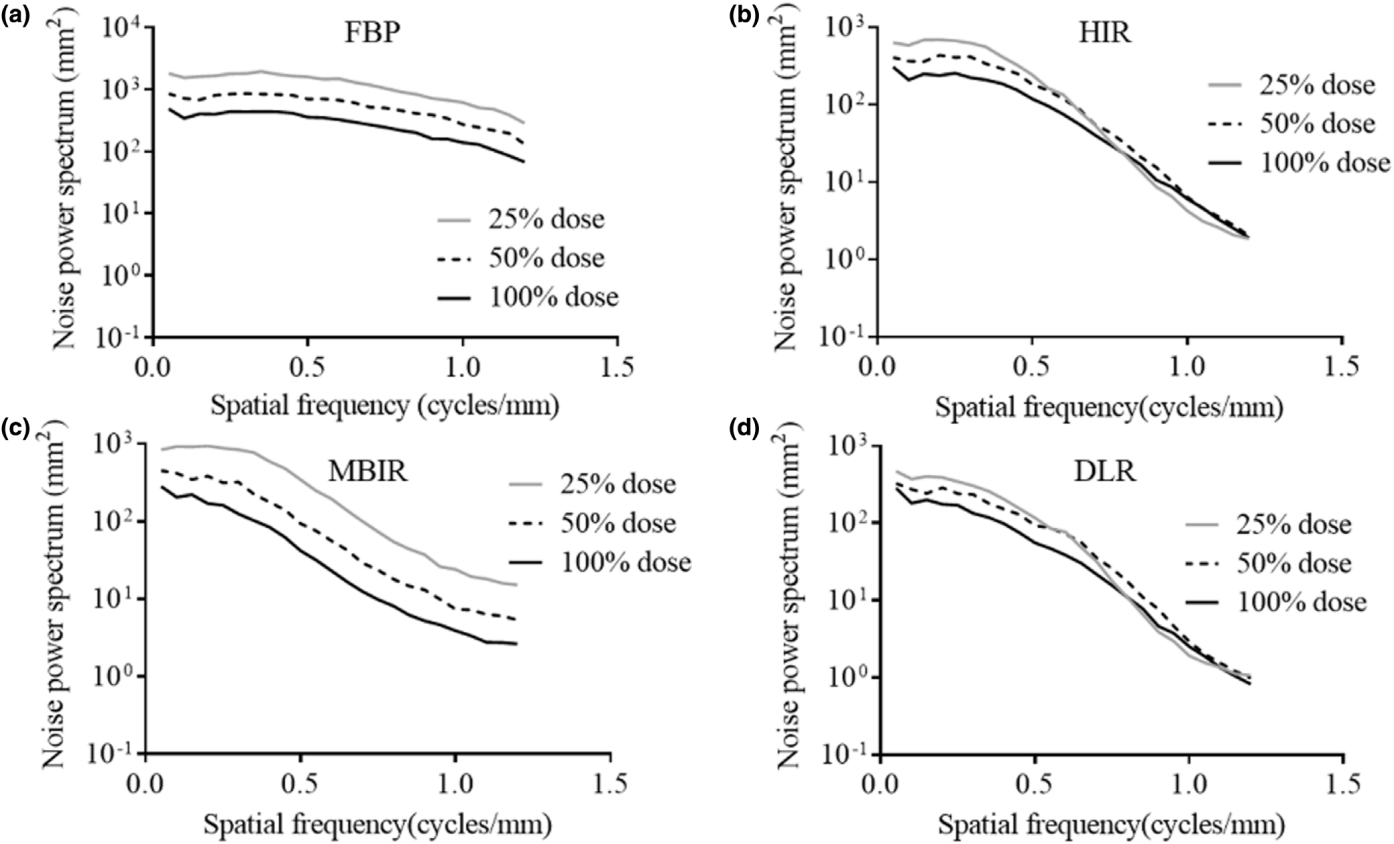
The noise power spectrum (NPS) curves of each reconstruction algorithm obtained at 100%, 50%, and 25% radiation doses. (a) Filtered back projection (FBP). (b) Hybrid iterative reconstruction (HIR). (c) Model‐based iterative reconstruction (MBIR). (d) Deep learning‑based reconstruction (DLR). As the radiation dose decreases, the NPS curves move upward in each reconstruction algorithm. The NPS curves in DLR were more similar to those in HIR than in the other reconstruction algorithms.

**Fig. 6:**
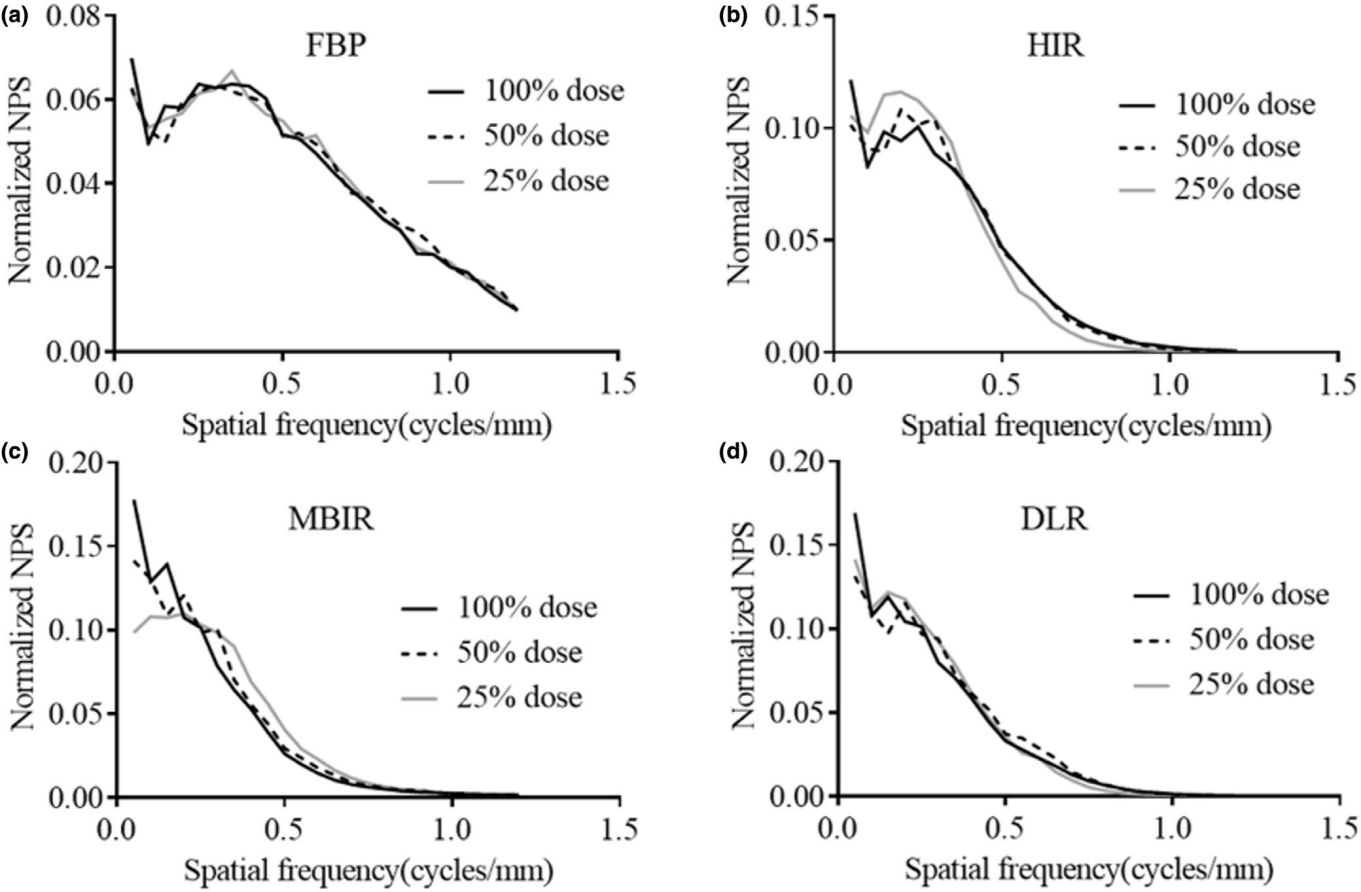
The normalized noise power spectrum (NPS) curves of each reconstruction algorithm obtained at 100%, 50%, and 25% radiation doses. (a) Filtered back projection (FBP). (b) Hybrid iterative reconstruction (HIR). (c) Model‐based iterative reconstruction (MBIR). (d) Deep learning‑based reconstruction (DLR). The shapes of the normalized NPS for DLR hardly varied at the different doses, while those of MBIR shifted slightly to a higher frequency as the radiation dose decreased.

Figure 5(a)–5(d) provides the NPS curves of each reconstruction algorithm obtained at the different radiation doses. Figure 6(a)–6(d) shows the normalized NPS.

### 3.3 Low contrast detectability

**Fig. 7:**
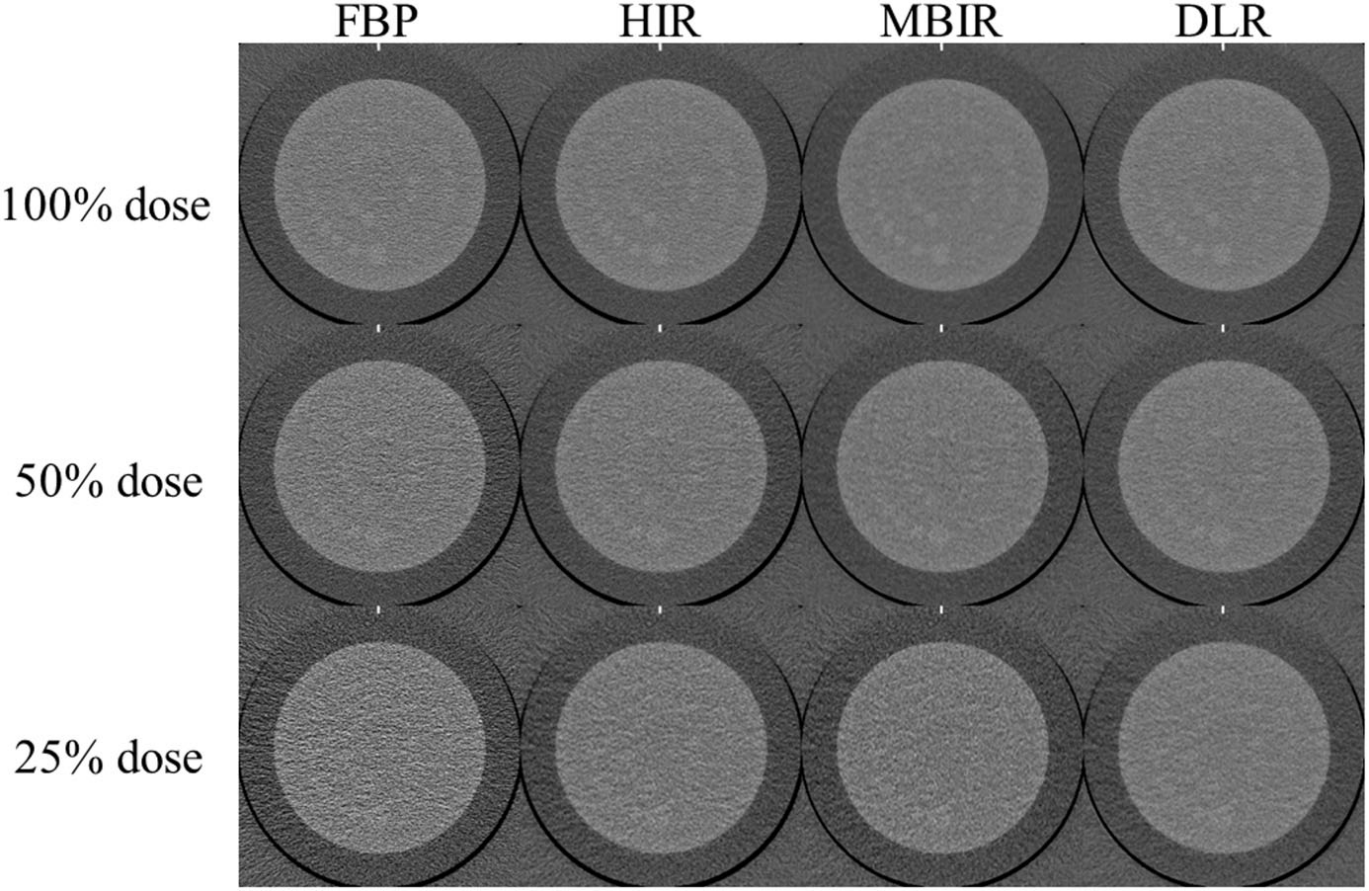
The clipped axial images (CTP515 module) of each reconstruction algorithm obtained at the 100%, 50%, and 25% radiation doses. Images were displayed with a window level and width of 60 and 250 HU.

Table 4 shows the detectable minimum diameter of each reconstruction algorithm at each relative radiation dose. As the radiation dose decreased, the detectable minimum diameters of the low‐contrast object increased at all reconstruction algorithms. A substantial interrater agreement was observed (*k* = 0.713). Figure 7 shows the clipped axial images of each reconstruction algorithm obtained at the different radiation doses.

**Table 4:** The detectable minimum diameters of the low‐contrast object at different radiation doses.

	Radiation dose	Reconstruction algorithm			
		FBP	HIR	MBIR	DLR
Detectable minimum diameter	100%	7.0	6.5	5.0	6.0
	50%	8.0	8.0	7.0	7.5
	25%	9.0	9.0	9.0	8.0
mm					

Abbreviations: DLR, deep learning‐based reconstruction; FBP, filtered back projection; HIR, hybrid iterative reconstruction; MBIR, model‐based iterative reconstruction.

### 3.4 Attenuation profile curves

**Fig. 8:**
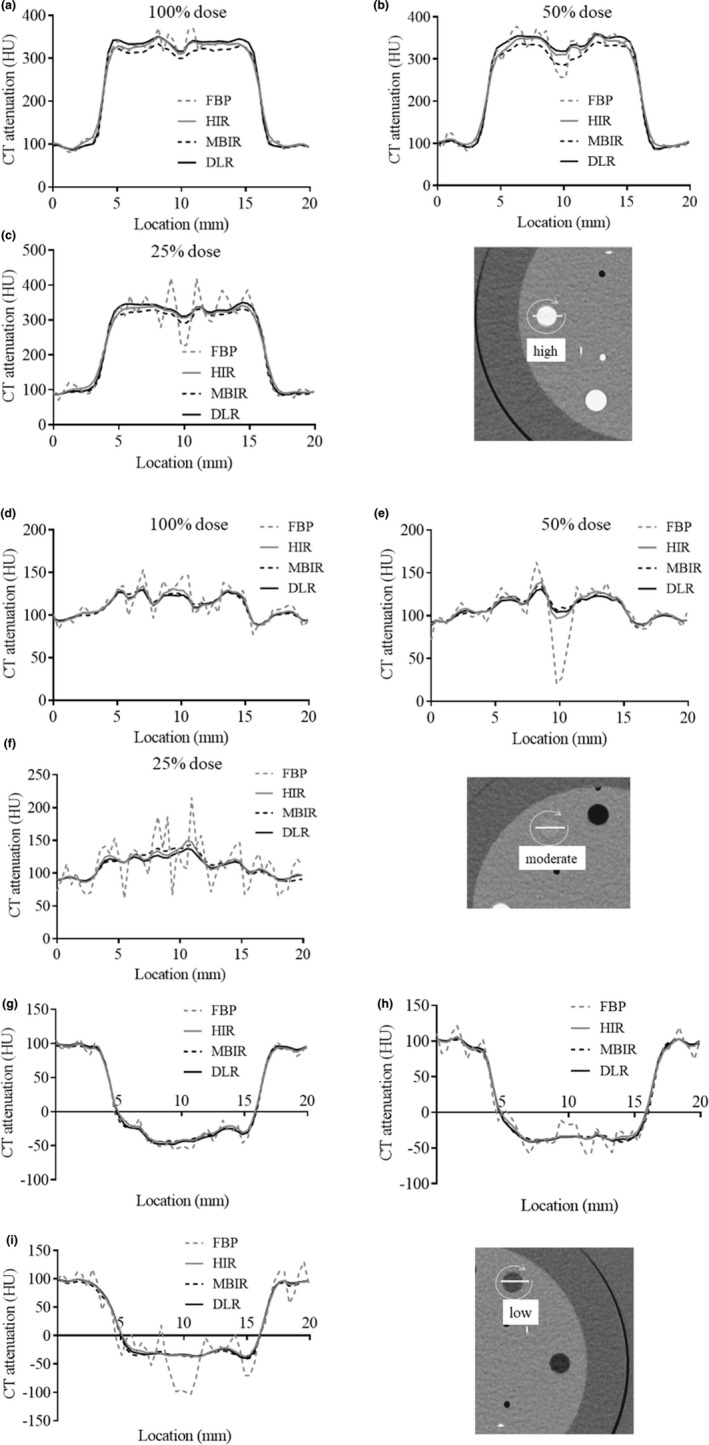
Attenuation profile curves (APCs) for all reconstruction algorithms obtained at 100%, 50%, and 25% radiation doses. The APCs of the (a–c) high‐signal object, (d–f) moderate‐signal object, and (g–i) low‐signal object obtained at 100%, 50%, and 25% radiation doses, respectively. The APCs were affected by the applied reconstruction algorithm. With the moderate‐signal object, the APCs had some amplitude and could be identified from the boundary of the background, except for the filtered back projection (FBP) algorithm. FBP, filtered back projection; HIR, hybrid iterative reconstruction; MBIR, model‐based iterative reconstruction; DLR, deep learning‐based reconstruction.

For the high‐signal objects, the APC of each reconstruction algorithm except for FBP was somewhat consistent within the signal test object under the lower radiation doses [Fig. 8(a)–5(c)]. With the moderate‐signal object [Fig. 8(d)–8(f)] (with the exception of FBP), the APCs of the signal test object had moderate amplitude, and it could be identified from the boundary of the background. For the low‐signal object [Fig. 8(g)–8(i)] (with the exception of FBP), the APCs were nearly constant in the signal test object.

### 3.5 Spatial resolution

Table 5 shows the spatial frequency at 50% and 10% of MTF in each reconstruction algorithm generated with the different radiation doses. The spatial frequencies at 50% and 10% of the MTF of DLR scanned with the 100% radiation dose were 0.62 and 1.00 cycles/mm, respectively. When the radiation dose was decreased, the spatial frequency at the 50% value of the MTF of DLR declined slightly, but that of the 10% value did not change significantly (50% and 25% doses, 0.98 and 0.99 cycles/mm, respectively).

**Table 5:** Spatial frequency at 50% and 10% of MTF at different radiation doses.

Radiation dose	MTF	Reconstruction algorithm			
		FBP	HIR	MBIR	DLR
100%	50%	0.47 (0.002)	0.37 (0.001)	0.56 (0.003)	0.62 (0.004)
	10%	0.97 (0.014)	0.72 (0.005)	1.05 (0.025)	1.00 (0.021)
50%	50%	0.46 (0.003)	0.34 (0.002)	0.53 (0.002)	0.59 (0.003)
	10%	1.01 (0.015)	0.65 (0.001)	1.05 (0.019)	0.98 (0.013)
25%	50%	0.49 (0.001)	0.31 (0.001)	0.51 (0.002)	0.55 (0.002)
	10%	1.02 (0.014)	0.59 (0.002)	1.02 (0.013)	0.99 (0.012)
Cycles/mm (SE)					

Abbreviations: DLR, deep learning‐based reconstruction; FBP, filtered back projection; HIR, hybrid iterative reconstruction; MBIR, model‐based iterative reconstruction; MTF, modulation transfer function; SE, standard error.

## 4 DISCUSSION

The results of these experiments demonstrated that the DLR maintained the same image noise magnitude and spatial resolution at all radiation doses with less change in signal visibility than the commercially available reconstruction methods analyzed. The DLR algorithm may provide image quality benefits to U‐HRCT platforms over those of the other techniques. It is notable that the image noise in the DLR was relatively consistent across a wide range of radiation doses. In the comparison of the DLR and MBIR algorithms, the image noise was higher in DLR than in MBIR at the highest radiation dose. However, at the lowest radiation dose, the image noise in DLR was lower than that in MBIR. We speculate that the MBIR algorithm might sacrifice de‐noising in order to maintain the spatial resolution at lower radiation doses, whereas DLR provided a better tradeoff in terms of noise versus dose. Therefore, for the range of radiation doses investigated, the DLR algorithm can improve image noise performance at low CT doses, which is critical for the larger matrix utilized in U‐HRCT.

The frequency characteristics of the image noise in DLR were similar to those in HIR although the training image of DLR is generated using a high‐dose MBIR image. The HIR image is reconstructed first and used internally as an input image for the DLR image process.^16^ Therefore, the shape of the NPS of the DLR image might be similar to those of HIR.

The shape of the normalized NPS for DLR minimally varied at the different radiation doses, while that of MBIR shifted slightly to a higher frequency as the radiation dose decreased. It is notable that the normalized NPS of DLR did not vary according to the radiation dose, and thus DLR can provide de‐noising without a change in noise texture compared to MBIR. The image noise frequency characteristics in MBIR observed in this study contrasted with findings of previous studies.^18^, ^19^ We believe these differences may be due to photon starvation from the low doses utilized in this study, the number of channels in the CT system (conventional CT, U‐HRCT: approximately 890 channels, 1792 channels, respectively), and the increased phantom size (circular phantoms of 15.0 and 21.5 cm diameter, in previous studies, versus an oval phantom of 25 × 35 cm in the present study). The MBIR performance in this study appears be much more dose‐dependent than DLR performance. Therefore, DLR appears more likely to provide superior reconstruction capabilities under the necessary low‐dose conditions of U‐HRCT. We thus consider DLR to be useful for dynamic abdominal CT on U‐HRCT, which tends to have excessive noise.

In terms of low‐contrast detectability, DLR showed the second smallest detectable diameter of a low contrast object after MBIR at the 100% and 50% doses. At a 25% dose, DLR showed the smallest detectable diameter of a low‐contrast object among the reconstruction algorithms. Thus, DLR demonstrated superior performance in low contrast detectability at reduced dose compared to the other reconstruction algorithms. This trend may reflect the relative consistency of the image noise magnitude across a wide range of radiation doses. Therefore, DLR may be more useful under low‐dose conditions than other reconstruction algorithms.

DLR allowed less change in signal visibility compared to other reconstruction algorithms. In the conventionally used FBP and HIR algorithms, the reduction in X‐ray photons affects the visibility of the signal itself and the formation of the contour. Both edges of the signal test object were slightly blurred in the HIR compared to the other reconstruction algorithms under all three radiation doses. However, DLR allows less change in signal visibility even at a lower radiation dose. This may be due to the impact of the DLR algorithm being generated using high‐dose MBIR images which were obtained with many iterative computations as training data. We thus speculate that it may be possible to obtain accurate signal depictions that are not inferior to the MBIR images generated by a CT scanner.

In comparison with the other reconstruction algorithms, the spatial frequency at 10% of MTF of DLR was slightly higher than that of FBP (0.97 cycles/mm) and lower than that of MBIR (1.05 cycles/mm) scanned with the 100% radiation dose. As expected, the spatial frequency at 10% of the MTF curves of the DLR, FBP, and MBIR tended to be equivalent to each other after the radiation dose was decreased. However, the MTF of the HIR showed the lowest spatial resolution at each radiation dose. The 10% of the MTF with the DLR algorithm was not significantly inferior to that obtained with the MBIR algorithm. This is because the DLR algorithm is generated using a high‐dose MBIR image as a training image. As a result, the DLR algorithm has the MBIR feature of higher spatial resolution generated from an optical model, and its use achieves a spatial resolution that is not inferior to that of MBIR^16^, ^17^ Regarding HIR, at a radiation dose of 25%, HIR demonstrated superior noise reduction than MBIR. However, HIR significantly degraded the spatial frequency. We thus suspect that the low‐dose DLR images will be easier for radiologists to accept compared to low‐dose HIR and MBIR images in particular.

The modest increase in computational time for DRL yields substantial improvements in image quality over HIR with respect to spatial resolution and image noise, where the substantial additional time for MBIR only yields modest gains in spatial resolution (e.g., for reconstruction of images in the 20‐cm range, the computational time is approximately 20 s for FBP and HIR, approximately 9 min for MBIR, and approximately 90 s for DRL). In addition, for abdominal dynamic CT requiring multiphasic images, the requirement of hundreds of images multiplies the reconstruction time and affects the throughput in clinical practice.

Several limitations of this study should be acknowledged. First, we did not evaluate clinical images for individual abdominal dynamic CT on U‐HRCT. Rather, we focused on the quantitative evaluation of the behavior of the DLR algorithm at different dose settings assuming various body sizes on U‐HRCT. Further studies of diagnostic performances in clinical situations are needed. Second, this evaluation was limited to high resolution mode. Future analyses will be needed to evaluate superhigh‐resolution mode, which is expected to have higher image noise. We preliminarily evaluated this mode, though, with the lower dose setting that will partially imitate performance in the superhigh‐resolution mode. Further studies are needed to investigate DLR images using the superhigh‐resolution mode.

## 5 CONCLUSIONS

The present quantitative evaluations showed that the DLR performs more consistently at decreasing dose than MBIR, HIR, or FBP without extraordinary compromises in spatial resolution and low contrast detectability as compared with other reconstruction algorithms, and without a significant computation penalty. In particular, at lower radiation doses, DRL quantitatively performed better than MBIR and is expected to reduce image noise. For abdominal dynamic CT on U‐HRCT, DLR may be a promising tool to compensate for the increased image noise from smaller detectors in a larger matrix.

## Conflict of interest

Yoshiki Asayama and Akihiro Nishie are staff of joint research department in Kyushu University with Canon Medical systems corporation. The other authors have no conflict of interest.

## AUTHOR CONTRIBUTIONS

Study concepts: Shirasaka T. Study design: Shirasaka T, Kato T, Asayama Y. Data acquisition: Shirasaka T, Kojima T, Mikayama R, Hamasaki H, Ushijima Y. Quality control of data and algorithms: Funama Y, Asayama Y, Nishie A. Data analysis and interpretation: Shirasaka T, Sakai Y, Kondo M. Manuscript preparation: Shirasaka T. Manuscript editing: Funama Y, Kato T, Asayama Y, Nishie A. Manuscript review: All authors.

## Data Availability

Research data are not shared.
